# *Rothia dentocariosa* endophthalmitis following intravitreal injection—a case report

**DOI:** 10.1186/s12348-017-0142-3

**Published:** 2017-12-16

**Authors:** R. A. Hayes, H. Y. Bennett, S. O’Hagan

**Affiliations:** 1 University of Queensland School of Medicine, Brisbane, Australia; 2 Cairns Base Hospital, Cairns, Australia; 3 James Cook University College of Medicine and Dentistry, Cairns, Australia

**Keywords:** Anti-VEGF, Aflibercept, Endophthalmitis, Intraocular, Intravitreal injection, Rothia dentocariosa

## Abstract

**Purpose:**

This report describes the first recognised case of *Rothia dentocariosa* endophthalmitis following intravitreal injection.

**Case report:**

A 57-year-old indigenous Australian diabetic female developed pain, redness and decreased vision 3 days after intravitreal aflibercept injection to the right eye—administered for diabetic vitreous haemorrhage with suspected macular oedema and proliferative diabetic retinopathy. Examination revealed best corrected visual acuity (BCVA) of hand movements, ocular hypertension and marked anterior chamber inflammation. The left eye was unaffected but had a BCVA of 6/24 due to pre-existing diabetic retinopathy. Vitreous culture isolated *Rothia dentocariosa* as the organism responsible for the endophthalmitis. The following treatment with intraocular cephazolin, vancomycin and ceftazidime, topical ciprofloxacin and gentamicin and systemic ciprofloxacin, the patient underwent vitrectomy. Nine weeks after onset, the patient’s BCVA had improved to 6/36, and fundal examination revealed extensive retinal necrosis.

**Conclusion:**

*Rothia dentocariosa* is presented as a rare cause of endophthalmitis following intravitreal injection and reports the appearance of ‘pink hypopyon’ previously observed with other organisms. Its identification also demonstrates the risk of oral bacterial contamination during intraocular injections. Vigilance with strategies to minimise bacterial contamination in the peri-injection period are important. Further research to identify additional techniques to prevent contamination with oral bacteria would be beneficial, including whether a role exists for patients wearing surgical masks during intravitreal injections.

## Case

A 57-year-old indigenous Australian diabetic female presented with decreased visual acuity, pain and redness in the right eye 3 days after intravitreal aflibercept injection (Eylea®—Regeneron, USA). The patient was a bilateral pseudophakic and had long-standing, high-risk proliferative diabetic retinopathy treated previously with bilateral intravitreal aflibercept and panretinal photocoagulation, as well as a vitrectomy for left tractional retinal detachment. No intraocular procedures had been performed in the affected eye 6 months prior to the most recent injection. The patient suffered with ischaemic heart disease, chronic kidney disease and refractory hypertension and was edentulous.

The indication for anti-VEGF therapy was right vitreous haemorrhage with suspicion of diabetic maculopathy, responsible for a pre-injection best corrected visual acuity (BCVA) of count fingers at 1 m. Routine right inferotemporal intravitreal injection was performed following preparation with povidone-iodine drops, application of topical chlorhexidine and facial draping. The injector wore a mask, and the procedure was followed by chloramphenicol drops QID, intended for 5 days.

Three days post-injection, the patient represented with right globe tenderness and declined BCVA—to hand movements. Intraocular pressure was 34 mmHg, and there was marked conjunctival injection, corneal oedema and anterior chamber inflammation with a 3.2-mm hypopyon. BCVA in the unaffected left eye was 6/24 with pinhole testing.

Systemic antibiotics were commenced with oral ciprofloxacin and intravenous vancomycin, and anterior chamber (AC) and vitreous sampling performed. Intracameral cephazolin 1 mg/0.1 mL, and intravitreal vancomycin 2 mg/0.1 mL and ceftazidime 2.25 mg/0.1 mL were administered following AC and vitreous taps. Post-operatively, ongoing therapy of oral ciprofloxacin 500 mg BD (adjusted for renal dysfunction, total of 14 doses), hourly ciprofloxacin 0.3% drops and hourly gentamicin 1.5% drops were implemented. Four days after the commencement of this regimen, topical prednisolone 1% was introduced second hourly. *Rothia dentocariosa* was isolated from the vitreous sample, while the anterior chamber sample demonstrated gram-positive cocci which could not be cultured. Antimicrobial sensitivities were not available due to the lack of qualifying data; however, discussion with a medical microbiologist recommended continuation of ciprofloxacin only—prompting cessation of gentamicin drops. Over the course of 4 weeks, the initial hypopyon transitioned into a layered hypopyon with a light pink pigmentation—similar to the appearance described in endophthalmitis caused by other organisms termed ‘pink hypopyon’ [1]. At 4 weeks, BCVA had improved to hand movements, the hypopyon had resolved and repeat AC and vitreous taps were performed showing no growth. Reinjections with intracameral cephazolin 1 mg/0.1 mL, and intravitreal vancomycin 2 mg/0.1 mL and ceftazidime 2.25 mg/0.1 mL were given. Seven weeks after the onset of endophthalmitis, right vitrectomy with intraocular lens explant, endolaser and epiretinal membrane peel was performed. Findings, intraoperatively at that time, demonstrated a widespread retinal ischaemia with necrosis superiorly and nasally, and she was left aphakic. At 9 weeks post-onset of endophthalmitis, she remained aphakic and her BCVA had improved to 6/36.

## Discussion

Coinciding with the expansion in application of intravitreal injections, post-injection endophthalmitis (PIE) is a potentially devastating complication—despite a low incidence rate (approximately 0.05% depending on meta-analysis) [2]. Mirroring post-surgical endophthalmitis, gram-positive cocci are the pre-dominant organism in PIE. Although coagulase-negative *staphylococcus* spp. (especially *Staphylococcus epidermidis)* are commonly identified, oral bacteria are often identified as causative organisms in PIE [2–5]. Several studies have investigated methods by which PIE can be prevented, focusing on strategies to minimise ocular contamination with oral microorganisms. Findings have supported decreased bacterial load with the use of povidone-iodine preparation [6], wearing of surgical masks by the injector and no-talking policies [6–8].

The oral microbiome is maintained with hundreds of species of bacteria, some of which are opportunistically infectious while others are seldom pathogenic [9, 10]. *Rothia dentocariosa* is a gram-positive bacteria that may be coccoid, diphtheroid or filamentous [11]. It is an oral commensal which is rarely of clinical significance [11]; however, isolated cases of *R. dentocariosa* causing serious infections have been reported in the literature—including endocarditis [12], osteomyelitis, septic arthritis [13], pneumonia [14] and peritonitis [15, 16]. To date, the only reported ocular infections with *R. dentocariosa* are a single case of endogenous endophthalmitis [17] and one case of superficial keratitis [18]. The *R. dentocariosa* endophthalmitis case reported by MacKinnon et al. (2001) was suspected to have arisen endogenously in a 73-year-old male, 1 month following AC reformation with viscoelastic injection. A case of potentially exogenous endophthalmitis caused by an unidentified *Rothia* species has also been reported, following perforating globe injury [19].

To our knowledge, we report the first case of *Rothia* sp. PIE in the literature, demonstrating its potential as a serious ophthalmological pathogen associated with intravitreal injections. The layered pink hypopyon present in this case has previously been described in endophthalmitis from a limited number of organisms—including *Serratia marcescens* and *Klebsiella pneumonia* [1]. Although the presence of concurrent vitreous haemorrhage in this case may be confounding, the presence of a pink hypopyon should prompt consideration for *Rothia* as a causative organism. In contrast to the only other case of confirmed *R. dentocariosa* endophthalmitis—of possible endogenous origin—exogenous inoculation during intravitreal injection was believed to be the mechanism of infection in this instance. Exogenous infection is thought likely due to the timing of the presentation (3 days post-intravitreal injection) and that the patient having no history of recent dental work as a potential source of haematogenous spread. The published cases of *R. dentocariosa* keratitis and *Rothia* sp. endophthalmitis could possibly have represented exogenous contamination but differed in that they were not reported in diabetics, the keratitis was superficial and the endophthalmitis occurred following delayed repair of globe rupture rather than from a sterile procedure.

## Conclusion

Despite all appropriate measures, PIE remains a potentially serious complication. To reduce this risk, contamination minimisation strategies should be evaluated. These include refraining from talking, wearing of surgical masks by injectors, the importance of proper sterile preparation and face draping, the role of antibiotics drops and counselling patients on avoiding autoinoculation after intraocular injection. Further studies investigating the role for patients wearing surgical masks to avoid contamination during intraocular injections and prior to application of the face drape would be of benefit.

## References

[1] Stefater JA, Borkar DS, Chodosh J (2015). Pink hypopyon in a patient with Serratia marcescens corneal ulceration. J Ophthalmic Inflamm Infect.

[2] Sachdeva MM, Moshiri A, Leder HA, Scott AW (2011) Endophthalmitis following intravitreal injection of anti-VEGF agents: long-term outcomes and the identification of unusual micro-organisms. J Ophthalmic Inflamm Infect. 10.1186/s12348-015-0069-5 10.1186/s12348-015-0069-5 PMC4710619 26758203

[3] Gentile RC, Shukla S, Shah M, Ritterband DC, Engelbert M, Davis A, D-N H (2014). Microbiological spectrum and antibiotic sensitivity in endophthalmitis. Ophthalmology.

[4] Rayess N, Rahimy E, Shah CP, Wolfe JD, Chen E, DeCroos FC, Storey P, Garg SJ, Hsu J (2016). Incidence and clinical features of post-injection endophthalmitis according to diagnosis. Br J Ophthalmol.

[5] Mccannel CA (2011). Meta-analysis of endophthalmitis after intravitreal injection of anti-vascular endothelial growth factor agents. Retina.

[6] Doshi RR, Leng T, Fung AE (2012). Reducing oral flora contamination of intravitreal injections with face mask or silence. Retina.

[7] Garg SJ, Dollin M, Hsu J, Storey P, Vander JF (2015). Effect of a strict ‘no-talking’ policy during intravitreal injection on post-injection endophthalmitis. Ophthalmic Surgery Lasers Imaging Retin.

[8] Wen JC, McCannel CA, Mochon AB, Garner OB (2011). Bacterial dispersal associated with speech in the setting of intravitreous injections. Arch Ophthalmol.

[9] Aas JA, Paster BJ, Stokes LN, Olsen I, Dewhirst FE (2005). Defining the normal bacterial flora of the oral cavity. J Clin Microbiol.

[10] Samaranayake L, Matsubara VH (2017). Normal oral flora and the oral ecosystem. Dent Clin N Am.

[11] von Graevenitz A (2004). Rothia dentocariosa: taxonomy and differential diagnosis. Clin Microbiol Infect.

[12] Shakoor S, Fasih N, Jabeen K, Jamil B (2011). Rothia dentocariosa endocarditis with mitral valve prolapse: case report and brief review. Infection.

[13] Ozan F, Öncel ES, Duygulu F, Çelik İ, Altay T (2015). Prosthetic hip joint infection caused by Rothia dentocariosa. Int J Clin Exp Med.

[14] Schiff MJ, Kaplan MH (1987). Rothia dentocariosa pneumonia in an immunocompromised patient. Lung.

[15] Ramanan P, Barreto JN, Osmon DR, Tosh PK (2014). Rothia bacteremia: a 10-year experience at Mayo Clinic, Rochester, Minnesota. J Clin Microbiol.

[16] Ergin C, Sezer MT, Agalar C, Katirci S, Demirdal T, Yayli G (2000). A case of peritonitis due to Rothia dentocariosa in a CAPD patient. Perit Dial Int.

[17] MacKinnon MM, Amezaga MR, MacKinnon JR (2001). A case of Rothia dentocariosa endophthalmitis. Eur J Clin Microbiol Infect Dis.

[18] Morley AMS, Tuft SJ (2006). Rothia dentocariosa isolated from a corneal ulcer. Cornea.

[19] Partner AM, Bhattacharya S, Scott RAH, Stavrou P (2006). Rothia genus endophthalmitis following penetrating injury in a child. Eye (Lond).

